# Symptoms of Anxiety and Depression Among Adults with Arthritis — United States, 2015–2017

**DOI:** 10.15585/mmwr.mm6739a2

**Published:** 2018-10-05

**Authors:** Dana Guglielmo, Jennifer M. Hootman, Michael A. Boring, Louise B. Murphy, Kristina A. Theis, Janet B. Croft, Kamil E. Barbour, Patricia P. Katz, Charles G. Helmick

**Affiliations:** ^1^Division of Population Health, National Center for Chronic Disease Prevention and Health Promotion, CDC; ^2^Oak Ridge Institute for Science and Education, Oak Ridge, Tennessee; ^3^University of California, San Francisco.

An estimated 54.4 million (22.7%) U.S. adults have doctor-diagnosed arthritis (*1*). A report in 2012 found that, among adults aged ≥45 years with arthritis, approximately one third reported having anxiety or depression, with anxiety more common than depression (*2*). Studies examining mental health conditions in adults with arthritis have focused largely on depression, arthritis subtypes, and middle-aged and older adults, or have not been nationally representative (*3*). To address these knowledge gaps, CDC analyzed 2015–2017 National Health Interview Survey (NHIS) data* to estimate the national prevalence of clinically relevant symptoms of anxiety and depression among adults aged ≥18 years with arthritis. Among adults with arthritis, age-standardized prevalences of symptoms of anxiety and depression were 22.5% and 12.1%, respectively, compared with 10.7% and 4.7% among adults without arthritis. Successful treatment approaches to address anxiety and depression among adults with arthritis are multifaceted and include screenings, referrals to mental health professionals, and evidence-based strategies such as regular physical activity and participation in self-management education to improve mental health.

NHIS is an ongoing, in-person, cross-sectional survey of the civilian, noninstitutionalized U.S. population. CDC analyzed combined NHIS data from 2015, 2016, and 2017 from the Sample Adult component of the survey, in which one adult is randomly selected from each family for whom additional information is collected. Response rates for the 3 years of surveys ranged from 53.0% to 55.2% and produced a 3-year sample of 93,442 participants. A randomly selected subset of approximately half of the sample adults (46,742) completed the Adult Functioning and Disability supplement over the 3-year period. Having arthritis was defined as a “yes” response to the question “Have you ever been told by a doctor or other health care professional that you have arthritis, rheumatoid arthritis, gout, lupus, or fibromyalgia?”

The Adult Functioning and Disability supplement included questions about symptoms of anxiety and depression. Respondents were classified as having symptoms of anxiety or depression if they reported the respective symptoms daily or weekly and responded that the last time they experienced symptoms, the intensity was “a lot” or “in between a little and a lot.”^†^ These definitions identified adults whose symptoms would likely meet *Diagnostic and Statistical Manual of Mental Disorders* (DSM-V) diagnostic criteria and also would be clinically managed, which are referred to in this report as “clinically relevant,” although these definitions are not clinical diagnoses.^§^^,^^¶^ The final unweighted sample sizes for those with arthritis who also reported whether they had anxiety or depression symptoms were 12,094 and 12,083, respectively.

Analyses accounted for the complex survey design, including the use of supplement file sampling weights so that weighted estimates derived from the sample were nationally representative. Age-standardized prevalences (using the 2000 projected U.S. population for persons aged 18–44, 45–64, and ≥65 years)** of symptoms of anxiety and depression were calculated for adults with and without arthritis and groups of those with arthritis who had selected sociodemographic and health-related characteristics. Prevalences of speaking with a mental health professional in the past 12 months and currently taking medications for symptoms of anxiety and depression^††^ also were calculated. T-tests were performed to assess statistical significance (p<0.05) when comparing differences.

During 2015–2017, age-standardized prevalences of symptoms of anxiety and depression among adults with arthritis were 22.5% (95% confidence interval [CI] = 20.8–24.3) and 12.1% (CI = 10.8–13.4), respectively. Prevalences among adults without arthritis were 10.7% (CI = 10.2–11.2) and 4.7% (CI = 4.4–5.0), respectively (Figure 1). When weighted estimates were applied, among adults with arthritis, an estimated 10.3 million reported symptoms of anxiety or depression; 4.9 million reported symptoms of anxiety only, 1.3 million reported symptoms of depression only, and 4.1 million reported symptoms of both.

**FIGURE 1:**
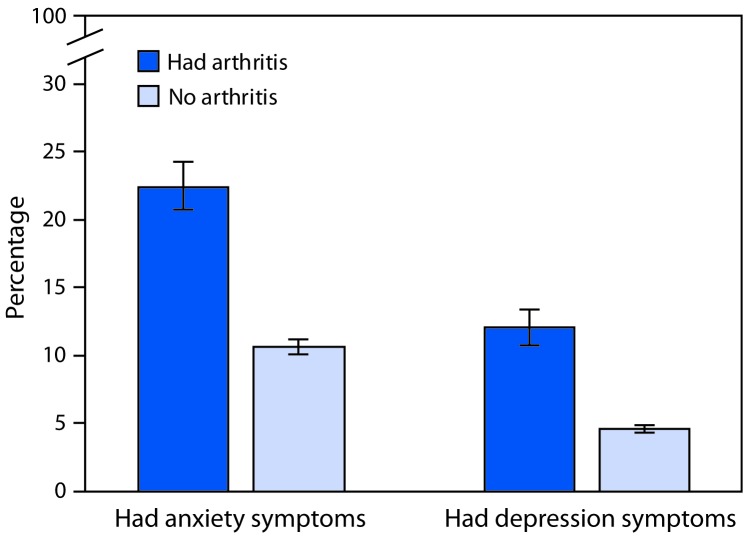
Age-standardized percentage* of adults reporting symptoms of anxiety and depression,^†^ by arthritis^§^ status — National Health Interview Survey, 2015–2017 * Estimates age-standardized to the 2000 projected U.S. population aged ≥18 years using three groups (18–44 years, 45–64 years, and ≥65 years). ^†^ Respondents were classified based on a frequency question (anxiety: “How often do you feel worried, nervous or anxious?” and depression: “How often do you feel depressed?”) and an intensity question (anxiety: “Thinking about the last time you felt worried, nervous or anxious, how would you describe the level of these feelings?” and depression: “Thinking about the last time you felt depressed, how depressed did you feel?”). Respondents were classified as having symptoms if they responded “daily” or “weekly” to the frequency question and “a lot” or “in between a little and a lot” to the intensity question. Respondents were classified as not having symptoms if they responded “daily” or “weekly” to the frequency question and “a little” to the intensity question, or if they responded “monthly,” “a few times a year,” or “never” to the frequency question. For each symptom, the remaining respondents were excluded from the analysis because their symptom status could not be identified. ^§^ Respondents were classified as having arthritis if they responded “yes” to “Have you ever been told by a doctor or other health care professional that you have arthritis, rheumatoid arthritis, gout, lupus, or fibromyalgia?”

Among adults with arthritis, age-specific prevalences of symptoms of anxiety and depression were higher among adults aged 18–44 years than among those aged ≥65 years; prevalence of symptoms of anxiety was also higher among adults with arthritis aged 18–44 years than adults with arthritis aged 45–64 years (Table). Age-standardized prevalences of symptoms of anxiety and depression were higher among women than among men; among those who were unemployed, unable to work, or disabled compared with employed adults; and among adults who reported their sexual identity as lesbian, gay, bisexual, or “other” than among those who reported being heterosexual. Symptom prevalences were lower among adults with higher educational and income-to-poverty ratios. Higher symptom prevalences were reported by adults with chronic pain and arthritis-attributable activity limitations, and prevalences increased with the number of co-occurring chronic conditions, increasing psychological distress, and declining self-rated health. Adults with arthritis who reported aerobic physical activity had lower anxiety and depression symptom prevalences than did inactive adults. Symptom prevalences also were higher among current cigarette smokers than among those who had never smoked.

**TABLE:** Age-standardized prevalence* of anxiety symptoms and depression symptoms**^†^** among adults aged ≥18 years with arthritis,**^§^** by selected characteristics — National Health Interview Survey, 2015–2017

Characteristic	Anxiety symptoms			Depression symptoms		
	Sample size	Unweighted no.	% (95% CI)^¶^	Sample size	Unweighted no.	% (95% CI)^¶^
**Overall**	12,094	2,039	22.5 (20.8–24.3)	12,083	1,304	12.1 (10.8–13.4)
**Sociodemographic**						
**Age group (yrs)^¶^**						
18–44	1,390	409	28.3 (25.2–31.5)	1,391	204	13.7 (11.5–16.2)
45–64	4,730	1,034	19.5 (18.1–21.0)	4,718	704	12.5 (11.4–13.8)
≥65	5,974	596	9.7 (8.8–10.7)	5,974	396	6.2 (5.5–7.1)
**Sex**						
Men	4,604	592	16.0 (13.8–18.5)	4,594	382	9.2 (7.4–11.4)
Women	7,490	1,447	26.9 (24.5–29.4)	7,489	922	14.0 (12.4–15.8)
Race/Ethnicity**						
White	9,195	1,556	23.9 (21.8–26.1)	9,187	953	12.0 (10.5–13.6)
Black	1,392	189	17.8 (13.8–22.7)	1,389	152	13.6 (9.6–18.8)
Hispanic	921	190	20.3 (15.9–25.7)	920	131	12.4 (9.4–16.0)
Asian	285	26	10.6 (4.7–22.0)	285	20	3.4 (1.8–6.1)
American Indian/Alaska Native	87	24	21.7 (10.7–39.1)	87	17	15.4 (7.9–27.7)
Other/Multiple race	214	54	32.3 (21.6–45.4)	215	31	17.4 (8.5–32.1)
**Education**						
Less than high school graduate	1,784	379	27.9 (22.9–33.4)	1,780	282	19.4 (15.4–24.2)
High school graduate or equivalent	3,347	549	23.1 (19.5–27.2)	3,352	346	12.9 (10.1–16.3)
Technical school/Some college	3,816	676	23.8 (21.2–26.7)	3,804	439	11.8 (9.9–13.9)
College degree or higher	3,108	426	17.9 (15.1–21.2)	3,107	234	8.6 (6.8–10.8)
**Employment status**						
Employed/Self-employed	4,453	643	17.0 (14.9–19.2)	4,453	323	7.0 (5.9–8.3)
Unemployed	250	86	32.9 (24.9–42.1)	250	56	19.6 (12.8–28.9)
Unable to work/Disabled	6,916	1,197	36.6 (32.0–41.4)	6,910	863	25.9 (21.7–30.7)
Other	472	113	26.9 (21.1–33.7)	467	62	14.9 (10.4–20.9)
**Income-to-poverty ratio (IPR)^††^**						
Poor (IPR<100%)	1,796	605	37.0 (32.5–41.7)	1,792	449	27.1 (22.9–31.9)
Near poor (100%≤IPR<125%)	714	159	28.6 (21.4–37.0)	715	99	16.2 (10.6–23.9)
Low income (125%≤IPR<200%)	1,891	319	27.0 (22.2–32.5)	1,889	206	12.7 (9.7–16.5)
Middle income (200%≤IPR<400%)	3,579	542	21.1 (17.7–24.9)	3,579	336	11.2 (9.2–13.5)
High income (IPR≥400%)	4,115	414	15.3 (12.9–18.0)	4,108	214	6.0 (4.6–7.8)
**Sexual identity**						
Heterosexual	11,625	1,903	21.7 (20.0–23.6)	11,614	1,205	11.6 (10.3–13.0)
Lesbian/Gay/Bisexual/Other	323	104	36.9 (29.0–45.5)	323	76	21.3 (15.7–28.2)
**Health-related **						
**BMI (kg/m^2^)**						
Underweight/Healthy weight (<25)	3,080	519	24.2 (20.9–27.8)	3,078	312	11.6 (9.5–14.0)
Overweight (25 to <30)	3,866	549	16.6 (14.0–19.6)	3,865	350	9.4 (7.5–11.8)
Obese (≥30)	4,797	912	25.6 (22.8–28.6)	4,785	615	14.2 (12.1–16.5)
**No. of co-occurring chronic conditions^§§^**						
0	3,031	435	18.0 (15.6–20.6)	3,029	234	8.1 (6.6–9.8)
1–2	6,358	979	23.6 (20.8–26.5)	6,355	590	13.0 (11.0–15.3)
≥3	2,705	625	40.2 (33.5–47.2)	2,699	480	27.9 (22.0–34.6)
**Psychological distress^¶¶^**						
None/Mild (K6≤4)	8,495	388	6.7 (5.4–8.3)	8,487	122	1.6 (1.1–2.3)
Moderate (5≤K6≤12)	2,719	997	40.8 (37.4–44.2)	2,721	594	20.1 (17.5–23.0)
Severe (K6≥13)	817	635	81.9 (77.4–85.7)	813	573	67.6 (60.4–74.0)
**Self-rated health**						
Excellent/Very good	4,669	424	15.3 (12.9–18.1)	4,659	193	5.5 (4.0–7.5)
Good	4,037	593	20.5 (17.9–23.3)	4,041	339	10.3 (8.5–12.4)
Fair/Poor	3,385	1,020	36.8 (33.0–40.7)	3,380	772	25.1 (21.8–28.8)
Chronic pain***						
No	6,317	592	14.7 (12.6–17.1)	6,310	299	6.0 (4.8–7.6)
Yes	5,765	1,446	31.2 (28.4–34.0)	5,769	1,005	18.7 (16.6–21.0)
**Arthritis-attributable activity limitations^†††^**						
No	6,667	692	15.6 (13.7–17.7)	6,666	383	7.3 (6.0–8.8)
Yes	5,423	1,346	32.5 (29.5–35.7)	5,413	921	18.9 (16.6–21.5)
**Aerobic physical activity level^§§§^**						
Active	4,658	630	18.7 (16.3–21.3)	4,657	330	8.2 (6.9–9.9)
Insufficient	2,708	475	24.5 (20.9–28.5)	2,703	296	12.3 (10.1–15.0)
Inactive	4,572	899	26.8 (23.3–30.6)	4,567	655	18.1 (14.9–21.9)
**Smoking status^¶¶¶^**						
Current smoker	1,987	599	31.8 (28.3–35.5)	1,985	430	21.5 (18.6–24.8)
Former smoker	4,080	604	25.2 (21.4–29.5)	4,077	379	10.9 (8.6–13.8)
Never smoker	6,016	832	17.5 (15.3–19.8)	6,009	494	8.6 (7.1–10.3)
Binge drank alcohol in past 30 days****						
No	10,870	1,777	22.3 (20.3–24.3)	10,856	1,157	11.7 (10.3–13.2)
Yes	1,074	227	23.9 (20.2–28.0)	1,075	126	13.5 (10.5–17.3)
**Have usual place for care**						
No	551	108	22.1 (16.5–28.9)	553	69	11.0 (7.8–15.2)
Yes	11,543	1,931	22.5 (20.7–24.4)	11,530	1,235	12.1 (10.8–13.6)

**Abbreviations:** BMI = body mass index; CI = confidence interval; K6 = Kessler-6 score.

* Estimates were age-standardized to the 2000 projected U.S. population aged ≥18 years using three groups (18–44 years, 45–64 years, and ≥65 years).

^†^ Respondents were classified based on a frequency question (anxiety: “How often do you feel worried, nervous or anxious?” and depression: “How often do you feel depressed?”) and an intensity question (anxiety: “Thinking about the last time you felt worried, nervous or anxious, how would you describe the level of these feelings?” and depression: “Thinking about the last time you felt depressed, how depressed did you feel?”). Respondents were classified as having symptoms if they responded “daily” or “weekly” to the frequency question and “a lot” or “in between a little and a lot” to the intensity question. Respondents were classified as not having symptoms if they responded “daily” or “weekly” to the frequency question and “a little” to the intensity question, or if they responded “monthly,” “a few times a year,” or “never” to the frequency question. For each symptom, the remaining respondents were excluded from the analysis because their symptom status could not be identified.

^§^ Respondents were classified as having arthritis if they responded “yes” to the question “Have you ever been told by a doctor or other health care professional that you have arthritis, rheumatoid arthritis, gout, lupus, or fibromyalgia?” Respondents with the values, “don’t know,” “missing,” or “refused,” for the arthritis case-finding question were excluded from the analytic sample.

^¶^ Age group percentages are age-specific, and all other percentages are age-standardized.

** Persons who identified as Hispanic might be of any race. Persons who identified with a racial group were all non-Hispanic.

^††^ Income-to-poverty ratio was calculated using income data generated using multiple imputation.

^§§^ Among nine chronic conditions (asthma, cancer, chronic obstructive pulmonary disease, diabetes, heart disease, hepatitis, hypertension, kidney disease, and stroke).

^¶¶^ Psychological distress was classified using the Kessler-6 scale, a 24-point scale capturing the presence and severity of nonspecific psychological distress symptoms in the past 30 days, as none/mild (Kessler-6 score [K6]≤4), moderate (5≤K6≤12), and severe (K6≥13).

*** Respondents were classified as having chronic pain if they reported having pain most days or every day in the past 3 months.

^†††^ Respondents were classified as having arthritis-attributable activity limitations if they responded “yes” to the question “Are you now limited in any way in any of your usual activities because of arthritis or joint symptoms?”

^§§§^ Respondents were classified as active based on the 2008 Physical Activity Guidelines for Americans if they reported ≥150 minutes of moderate intensity leisure time aerobic physical activity per week, insufficiently active if they reported 1–149 minutes, and inactive if they reported zero minutes. Reported vigorous intensity physical activity minutes were counted double and added to moderate intensity physical activity minutes.

^¶¶¶^ Respondents were classified as ever having smoked if they had smoked at least 100 cigarettes in their lifetime.

**** Binge drinking was defined as consuming five or more drinks (men) or four or more drinks (women) over a 2-hour period.

Taking medications was less common among arthritis patients who had anxiety symptoms (44.3%; CI = 40.4–48.3) than among those with symptoms of depression (57.7%; CI = 52.4–62.9) (Figure 2). Speaking with a mental health professional in the past 12 months was reported by 34.3% (CI = 30.3–38.1) of arthritis patients with anxiety symptoms and 42.8% (CI = 37.7–48.1) of those with symptoms of depression.

**FIGURE 2:**
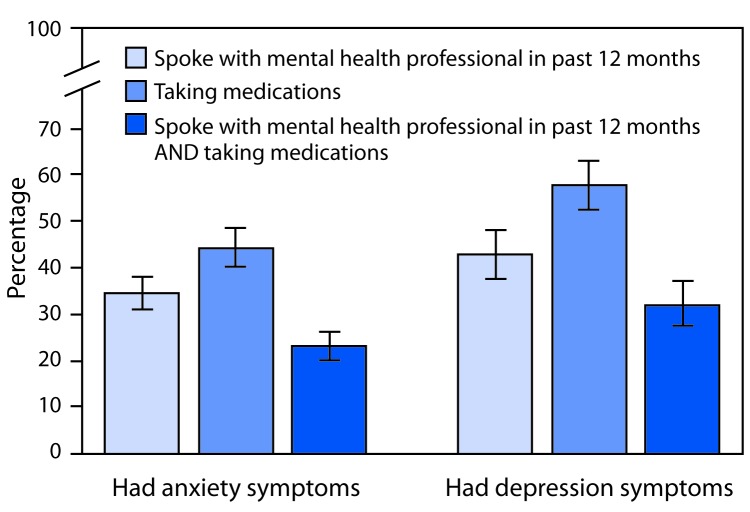
Age-standardized percentage* of adults with arthritis^†^ reporting treatment for anxiety symptoms or depression symptoms,^§^ by type of treatment^¶^^,^** — National Health Interview Survey, 2015–2017 * Estimates were age-standardized to the 2000 projected U.S. population aged ≥18 years using three groups (18–44 years, 45–64 years, and ≥65 years). ^†^ Respondents were classified as having arthritis if they responded “yes” to the question “Have you ever been told by a doctor or other health care professional that you have arthritis, rheumatoid arthritis, gout, lupus, or fibromyalgia?” ^§^ Respondents were classified based on a frequency question (anxiety: “How often do you feel worried, nervous or anxious?” and depression: “How often do you feel depressed?”) and an intensity question (anxiety: “Thinking about the last time you felt worried, nervous or anxious, how would you describe the level of these feelings?” and depression: “Thinking about the last time you felt depressed, how depressed did you feel?”). Respondents were classified as having symptoms if they responded “daily” or “weekly” to the frequency question and “a lot” or “in between a little and a lot” to the intensity question. Respondents were classified as not having symptoms if they responded “daily” or “weekly” to the frequency question and “a little” to the intensity question, or if they responded “monthly,” “a few times a year,” or “never” to the frequency question. For each symptom, the remaining respondents were excluded from the analysis because their symptom status could not be identified. ^¶^ Spoke with a mental health professional in the past 12 months was defined by the question “During the past 12 months, have you seen or talked to a mental health professional such as a psychiatrist, psychologist, psychiatric nurse, or clinical social worker?” ** Taking medications was defined as responding “yes” to the question “Do you take medication for these feelings?” (anxiety) or “Do you take medication for depression?”

## Discussion

This report presents national estimates of clinically relevant symptoms of anxiety and depression among U.S. adults with arthritis. In the United States, an estimated 10.3 million adults with arthritis reported symptoms of anxiety, depression, or both. Prevalences of symptoms of anxiety and depression were substantially higher among adults with arthritis than among those without arthritis, and among adults with arthritis, were substantially higher among younger adults than among older adults.

Similar to previous studies of adults with arthritis overall and for arthritis subtypes (e.g., osteoarthritis or rheumatoid arthritis) (*2*,*4*,*5*), the prevalence of anxiety symptoms exceeded that of symptoms of depression. Despite this, adults with anxiety symptoms less commonly reported taking medications for their symptoms than did those with symptoms of depression; the prevalences among those with either anxiety or depression symptoms were not statistically different for speaking with a mental health professional.

Those with arthritis who were unable to work or were disabled reported higher prevalences of symptoms of anxiety and depression than those who were employed, and adults aged 18–64 years reported higher prevalences of each than those aged ≥65 years. Mental health conditions (i.e., depression, anxiety, or emotional problems) and arthritis were previously reported as two of the top three causes of work disability among adults aged 18–64 years in 2011–2013 (*6*). Concerted efforts to improve arthritis and mental health outcomes could help reduce work disability. Adults with any work disability and employers can consult the Job Accommodation Network, a free service that provides extensive resources on job accommodations and Americans with Disabilities Act compliance.^§§^

Among adults with arthritis and chronic pain, symptoms of anxiety and depression were reported among 31.2% and 18.7%, respectively. A potential link exists between chronic pain and anxiety or depression, which might complicate physical and mental health management for persons with arthritis (*7*). Having arthritis has been associated with reduced adherence to treatment for depression (*8*), and in 2000–2001, nearly one in five surveyed persons with arthritis and major depression reported suicidal ideation within the past year (*9*). In clinic-based rheumatic disease studies, both anxiety and depression were associated with reduced response to treatment (*10*) and poorer quality of life (*4*). In addition, the National Institute of Mental Health estimates that only half of persons with a mental health condition receive treatment^¶¶^; the current analysis suggests that treatment prevalence among adults with arthritis might be similar or lower, especially for anxiety.

The occurrence of widespread anxiety and depression symptoms among adults with arthritis points to an unmet need that health care providers can address. The U.S. Preventive Services Task Force recommends depression screening for all adults***; the Substance Abuse and Mental Health Services Administration encourages screening persons of all ages for anxiety and depression^†††^; and The Guide to Community Preventive Services recommends collaborative care for depression.^§§§^ The National Pain Strategy encourages addressing chronic pain conditions like arthritis with integrated care and self-management education.^¶¶¶^ Health care providers can refer their arthritis patients to evidence-based programs like the Chronic Disease Self-Management Program, which has benefits including sustained reductions in depression, fatigue, and pain, and increases in aerobic activity, self-efficacy, and self-rated health.****^,††††^ Providers can also suggest physical activity, which can improve symptoms of clinical anxiety and depression and can be as effective as medication or therapy for anxiety and depression.^§§§§^ Even those who do not meet the full recommended federal guidelines can still receive physical and psychological benefits from physical activity.^¶¶¶¶^

The findings in this report are subject to at least three limitations. First, NHIS data are self-reported, and some characteristics might be susceptible to recall and social desirability biases and underreporting because of potential stigma. Second, symptoms of anxiety and depression are not equivalent to clinical diagnoses; the questions ascertaining symptoms have no time frame, the intensity question only refers to the most recent episode, and cases cannot be validated. Finally, NHIS data are cross-sectional, so the temporal sequence of arthritis, anxiety, and depression, and other characteristics cannot be determined.

Symptoms of anxiety and depression are common among U.S. adults with arthritis. Whereas groups of adults with arthritis who have the highest prevalences of symptoms of anxiety and depression might be high treatment priorities, the high overall prevalence of each indicator compared with those among adults without arthritis suggests that all adults with arthritis would benefit from mental health screening. Health care providers can help their arthritis patients by screening and considering treating or referring adults with symptoms to mental health professionals or self-management education programs, and encouraging physical activity, which is an effective nonpharmacologic strategy that can help reduce the symptoms of anxiety and depression, improve arthritis symptoms, and promote better quality of life.

**Summary**

**What is already known about this topic?**

In adults with arthritis, anxiety and depression are associated with poorer overall health and quality of life.

**What is added by this report?**

Among adults with arthritis, 22.5% reported symptoms of anxiety and 12.1% reported depression. Anxiety and depression symptoms were more common among younger adults, those with chronic pain or comorbid chronic conditions, and those unable to work or who were disabled.

**What are the implications for public health practice?**

The high prevalence of symptoms of anxiety and depression among adults with arthritis warrants awareness, screening, and subsequent treatment of these conditions. Health care providers can refer patients to mental health professionals and self-management education programs, and encourage physical activity to reduce anxiety and depression symptoms and improve quality of life.

## Footnotes

* https://www.cdc.gov/nchs/nhis/data-questionnaires-documentation.htm.

† Respondents were classified based on a frequency question (anxiety: “How often do you feel worried, nervous or anxious?” and depression: “How often do you feel depressed?”) and an intensity question (anxiety: “Thinking about the last time you felt worried, nervous or anxious, how would you describe the level of these feelings?” and depression: “Thinking about the last time you felt depressed, how depressed did you feel?”). Respondents were classified as having symptoms if they responded “daily” or “weekly” to the frequency question and “a lot” or “in between a little and a lot” to the intensity question. Respondents were classified as not having symptoms if they responded “daily” or “weekly” to the frequency question and “a little” to the intensity question, or if they responded “monthly,” “a few times a year,” or “never” to the frequency question. For each symptom, the remaining respondents were excluded from the analysis because their symptom status could not be identified.

§ https://dsm.psychiatryonline.org/doi/full/10.1176/appi.books.9780890425596.dsm04.

¶ https://dsm.psychiatryonline.org/doi/full/10.1176/appi.books.9780890425596.dsm05.

** https://www.cdc.gov/nchs/data/statnt/statnt20.pdf.

†† Medication use for each of the anxiety or depression symptoms was ascertained from the question, “Do you take medication for these feelings?”

§§ https://askjan.org/.

¶¶ https://www.nimh.nih.gov/health/statistics/index.shtml.

***
https://www.uspreventiveservicestaskforce.org/Page/Document/RecommendationStatementFinal/depression-in-adults-screening1 .

††† https://www.integration.samhsa.gov/clinical-practice/screening-tools#bmb.

§§§ https://www.thecommunityguide.org/findings/mental-health-and-mental-illness-collaborative-care-management-depressive-disorders.

¶¶¶
https://iprcc.nih.gov/sites/default/files/HHSNational_Pain_Strategy_508C.pdf .

****
https://www.selfmanagementresource.com/programs/ .

§§§§
https://health.gov/paguidelines/second-edition/report/pdf/PAG_Advisory_Committee_Report.pdf .

¶¶¶¶
https://health.gov/paguidelines/guidelines/chapter4.aspx .

## References

[1] Barbour KE, Helmick CG, Boring M, Brady TJ. Vital signs: prevalence of doctor-diagnosed arthritis and arthritis-attributable activity limitation—United States, 2013–2015. MMWR Morb Mortal Wkly Rep 2017;66:246–53. 10.15585/mmwr.mm6609e1 28278145 PMC5687192

[2] Murphy LB, Sacks JJ, Brady TJ, Hootman JM, Chapman DP. Anxiety and depression among US adults with arthritis: prevalence and correlates. Arthritis Care Res (Hoboken) 2012;64:968–76. 22550055 10.1002/acr.21685

[3] Stubbs B, Aluko Y, Myint PK, Smith TO. Prevalence of depressive symptoms and anxiety in osteoarthritis: a systematic review and meta-analysis. Age Ageing 2016;45:228–35. 10.1093/ageing/afw001 26795974

[4] Anyfanti P, Gavriilaki E, Pyrpasopoulou A, Depression, anxiety, and quality of life in a large cohort of patients with rheumatic diseases: common, yet undertreated. Clin Rheumatol 2016;35:733–9. 10.1007/s10067-014-2677-0 24859781

[5] Sambamoorthi U, Shah D, Zhao X. Healthcare burden of depression in adults with arthritis. Expert Rev Pharmacoecon Outcomes Res 2017;17:53–65. 10.1080/14737167.2017.1281744 28092207 PMC5512931

[6] Theis KA, Roblin DW, Helmick CG, Luo R. Prevalence and causes of work disability among working-age U.S. adults, 2011–2013, NHIS. Disabil Health J 2018;11:108–15. 10.1016/j.dhjo.2017.04.010 28476583 PMC11131972

[7] Tunks ER, Crook J, Weir R. Epidemiology of chronic pain with psychological comorbidity: prevalence, risk, course, and prognosis. Can J Psychiatry 2008;53:224–34. 10.1177/070674370805300403 18478825

[8] DiMatteo MR, Lepper HS, Croghan TW. Depression is a risk factor for noncompliance with medical treatment: meta-analysis of the effects of anxiety and depression on patient adherence. Arch Intern Med 2000;160:2101–7. 10.1001/archinte.160.14.2101 10904452

[9] Fuller-Thomson E, Shaked Y. Factors associated with depression and suicidal ideation among individuals with arthritis or rheumatism: findings from a representative community survey. Arthritis Rheum 2009;61:944–50. 10.1002/art.24615 19565540

[10] Matcham F, Norton S, Scott DL, Steer S, Hotopf M. Symptoms of depression and anxiety predict treatment response and long-term physical health outcomes in rheumatoid arthritis: secondary analysis of a randomized controlled trial. Rheumatology (Oxford) 2016;55:268–78. 10.1093/rheumatology/kev306 26350486 PMC4710801

